# Vulnerability in Colorectal Cancer: Adjusted Gross Income and Geography as Factors in Determining Overall Survival in Colorectal Cancer: A Single-Center Study Across a Broad Income Inequality in an American Context

**DOI:** 10.3390/curroncol31120570

**Published:** 2024-12-03

**Authors:** Cataldo Doria, Patrick G. De Deyne, Papachristou Charalampos

**Affiliations:** 1 Capital Health Cancer Center, One Capital Way, Pennington, NJ 08534, USA; pdedeyne@gmail.com; 2 College of Science and Mathematics, Rowan University, 201 Mullica Hill Road, Glassboro, NJ 08028, USA; papachristou@rowan.edu

**Keywords:** CRC, vulnerability, disparity, socio economic status, zip codes, screening

## Abstract

**Introduction**: Regional differences in socioeconomic status (SES) are well known, and we believe that the use of geocoding (zip code) can facilitate the introduction of targeted interventions for underserved populations. This is a single-center, retrospective analysis of data extracted from the cancer registry at the Capital Health Cancer Center in Pennington, N. The Capital Health Cancer Center in central New Jersey primarily serves two counties, catering to a diverse patient population from a wide range of socioeconomic backgrounds. **Methods**: We abstracted 1269 consecutive cases of colorectal cancer (CRC) diagnosed and treated between 2000 and 2019 from the Cancer Registry of the Capital Health Cancer Center (CHCC). Using the definition of SES based on previously published work, and zip codes (geocoding), we created four SES levels. We stratified our subjects according to their stage at diagnosis, age at diagnosis, race, and ethnicity. The primary outcome variable was overall survival (OS). **Results**: There was a statistically significant difference in OS based on SES, with the highest overall survival (OS) in the high-SES group (47 months) and the shortest OS in the low and mid-low-SES groups (40.4 and 30 months, respectively). Subjects living in high-SES areas were predominantly white (88.2%) and diagnosed at a later age (mean of 68.9 years of age) compared to individuals who lived in a low-SES area, who were predominantly non-white (72.6%) and diagnosed somewhat earlier in life (65.1 years of age). White people were diagnosed later in life (70.9 years of age) compared to non-white populations, including Black (66.5), Asian (61.7), and Hispanic (58.5) (*p* = 0.001) populations, but this did not lead to a significant difference in OS (*p* = 0.56). Stage at diagnosis was a significant predictor of OS, but was unrelated to SES (*p* = 0.066). A Cox proportional hazard ratio (HR) model showed that the risk of dying from colorectal cancer decreases with a higher socioeconomic status (SES). Those from mid-high-SES backgrounds had a 19% lower risk (HR 0.81), and those from high-SES areas had a 45% lower risk (HR 0.55) compared to individuals from low-SES areas. **Conclusions**: The vulnerability of patients with CRC in central New Jersey is a complex issue, influenced by many different variables. Our results indicate that SES is the most critical factor affecting OS after being diagnosed with CRC.

## 1. Introduction

Addressing healthcare inequities in the US has been an ongoing effort for several years, and these issues have become even more pronounced since the COVID-19 pandemic [1,2]. An example of this disparity in the US can be illustrated by the incidence and mortality rate for colorectal cancer (CRC), which are 49.1 and 23 per 100,000 persons for AAs and 41.4 and 15.9 per 100,000 persons for Caucasian Americans [3]. Similar to many other illnesses, CRC disproportionally affects people from low socioeconomic backgrounds and racial minorities and the consensus is that increased access to care is paramount in curbing these inequities [4]. Implementing the most up-to-date screening strategies is an inherent component of achieving that goal [5]. Nationwide, the overall CRC screening prevalence among 50–75 year old individuals is 68.8% [6]. However, in people who have less than a high school education, have an annual household income of less than USD 35,000, have no health insurance, or do not have a primary care physician, the CRC screening rate ranges from 40 to 60%. These data suggest that preventive medicine interventions should be targeted to areas with a low socioeconomic status (SES, also called the physical area of intervention—PAOI) to reach vulnerable local populations. One way to identify a PAOI is through the combined use of zip codes and IRS annual tax information, which is also called geocoding [7]. Our main premise is that we first need to analyze our institutional clinical and outcome data for patients diagnosed with CRC before establishing a PAOI, and then develop appropriate strategies to reach vulnerable population in our regional area of service. Our hypothesis is supported by the results of our previous study that described regional health disparities in patients with pancreatic cancer [8].

We postulate that healthcare disparities can be addressed at a local and regional level by using geolocation (geocoding), which will have an impact on healthcare inequities. A similar approach was used previously in healthcare utilization [9] and, with modifications, in a study of CRC outcomes in rural areas [10]. Additionally, the role of SES and race in healthcare disparities has been previously investigated in the military, an organization where access to healthcare is expected, in a small study, and the authors did not find any differences in CRC outcomes based on race, claiming that access to proper care is an important determinant [11]. A more thorough analysis was performed using SEER data and it was found that socioeconomic determinants of health are by far the most important contributors to overall survival (OS), while the effect of race pointed to a more complex picture influenced by the CRC screening rate and access to care with highly specialized treatment modalities [12]. With respect to quality of life after CRC diagnosis, income, neighborhood, and SES have been suggested to be associated with overall outcomes [13]. The aim of our study was to use geocoding and combine this information with income data from individual tax returns to profile a regional sample of patients with CRC and to assess their outcomes (overall survival) based on SES, race, and ethnicity, thereby allowing us to identify a vulnerable segment of patients in our service area that is at a higher risk of experiencing worse outcomes once diagnosed with CRC. We believe that obtaining this information will allow regional medical centers to develop local strategies to reach these communities and to develop targeted CRC screening strategies.

## 2. Materials and Methods

Following the methodology of previous studies [8], the dataset for this research includes variables defined and standardized according to guidelines and specifications of the North American Association of Central Cancer Registries (NAACCR). Cancer stage was categorized based on the National Cancer Institute’s SEER Program, with stages classified as in situ, localized, regional (covering direct, nodal, or combined spread), or distant. This is a retrospective, single-site, cross-sectional study that leveraged data from our Institution’s cancer registry, which comprehensively records diagnoses, treatments, and follow-up information for each cancer patient treated in our hospital system. In an initial evaluation, we collected data from patients diagnosed with CRC from 2000 to 2019 residing in Mercer County, New Jersey, which has 12 municipalities and a suburban/urban population of 367,430 as of 2019. This approach captured 1269 cases of CRC, all treated at Capital Health, (subjects were an average of 68.7 years of age ± 14.3 (s.d.), and the sample consisted of 610 males and 659 females) [Table 1]. Using the patients’ zip code and tax return data associated with their zip code, we derived the socioeconomic status (SES) of the area in which each patient resides. Regarding the designation of race, we adopted strategies from previously published work on the matter [12].

The study protocol was reviewed and approved by the Capital Health Institutional Review Board.

Our aim was to determine whether SES and/or race had an impact on the overall survival, stage, and age at diagnosis of our patient population. We used a definition of SES based on previous research [14]: For income-based indicators, we used median household income, categorized into four groups using a priori cutoff points based on a government definition (Department of Housing and Urban Development 2013) defined as <60%, between 60 and 100%, between 100–140%, and >140% of median household income [15].

The median income for New Jersey in 2011 was USD 67,458 [16]. This approach led to the identification of four groups: low income of <60% of the median or USD 40,474; a mid-low income between 60 and 100% of the median (USD 40,474 and USD 67,458); mid-high income between 100 and 140% of the median (USD 67,458 and USD 94,441); and high income of >USD 94,441.

We extracted data from individual income tax returns, focusing on selected income and tax items by state, zip code, and adjusted gross income (AGI) for tax years 2011 to 2019 for the 21 zip codes in Mercer County, NJ. AGI information by zip code was unavailable on the Census website (https://data.census.gov, accessed on 30 October 2023) because it was not reported prior to 2011. Socioeconomic status (SES) appeared stable over the 10-year period, with only 5 out of the 21 zip codes (08618, 08611, 08619, 08560, and 08501) experiencing changes. These shifts affected the SES classifications of 407 patients: 325 moved from a mid-low to low SES, 61 moved from a mid-high to mid-low SES, and 21 moved from a high to mid-high SES. Our analyses, performed on data from each of these years, yielded virtually identical results in both qualitative and quantitative terms, as there was no substantial distinction between the two lowest SES categories, where the biggest shift occurred. Given that clinical and hospital data were collected for patients treated between 2000 and 2019, and that the financial data analyses for 2011–2019 showed no significant differences, we decided to use the SES designations based on the data from the 2011 tax year. This approach reasonably assumes that, similarly to the period 2011–2019, minimal SES changes occurred between 2000 and 2010, and that the 2011 SES data adequately represent the entire study period. The SES assignments for each of the 21 zip codes of interest are given in Table 2.

## 3. Statistical Analysis

The results are presented as the mean ± standard deviation (SD) for quantitative variables and as absolute frequencies and percentages for categorical variables. Continuous variables were compared across SES and racial groups using ANOVA, followed by pairwise comparisons using Tukey’s HSD (honestly significant difference) to adjust for multiplicity. OS was reported as the median and interquartile range (IQR) and differences across the groups were gauged using the Kruskal–Wallis test for non-normal data. Similarly, categorical variables were compared across groups using the chi-squared or Fisher’s exact test, where appropriate. Survival differences between SES and racial groups were assessed via Kaplan–Meier survival curves and log-rank tests and via multivariate Cox proportional hazard analysis to adjust for pertinent covariates such as race, sex, age, and cancer stage at diagnosis. Statistical analyses were performed using SAS^®^ (Cary, NC, USA) version 9.4 or above or software R version 4.2 or above (R Foundation for Statistical Computing, Vienna, Austria), with *p* < 0.05.

## 4. Results

The overall and general demographic characteristics of the participants are summarized in Table 1. The median follow-up time for the whole cohort was 37.1 months. With respect to the SES, Table 2 provides a summary of the most important zip codes in our service area and their classification according to median income and SES. For example, the median income in the zip code (08534) with the highest SES was 146,532 and the median income in the zip code (08608) with the lowest SES was 13,150, indicating a large income gap between the highest and the lowest zip codes.

As expected, stage at diagnosis was a critical determinant of OS, with patients diagnosed at stage 0 having a median (IQR) OS of 116 (86.143) months (n = 17), stage 1 having a median of 69 (31.133) months (n = 234), stage 2 having a median of 46 (18.83) months (n = 318), stage 3 having a median of 44 (21.93) months (n = 285), and stage 4 having a median of 10 (3.21) months (n = 258) with *p* < 0.001 (Kruskall–Wallis). When evaluating racial and ethnic differences [Table 3], we found that Caucasians were diagnosed at a later age (70.9 ± 14.1 years of age) compared to non-Caucasians (Black 66.5 ± 13.7, Asian 61.7 ± 10.9, Hispanic 58.5 ± 14.9 years of age), and this was statistically significant (*p* = 0.001, ANOVA); however, there was no statistically significant difference found in OS between Caucasian and non-Caucasian groups (*p* = 0.57), indicating that age at diagnosis is not correlated with race. In our analysis, neither race nor ethnicity were directly associated with OS despite having a differential distribution in our sample [Table 3]. Further clarification may be obtained by analyzing the racial composition of the four SES strata, which demonstrated racial and ethnic differences (Table 4, *p* < 0.001, ANOVA), with the majority of the white population living in an area of mid-high or high SES and the vast majority of the Black and Hispanic patients residing in low or mid-low-SES zip codes. These data indicate that SES (considering both geography and financial information) is associated with race.

**Figure 1:**
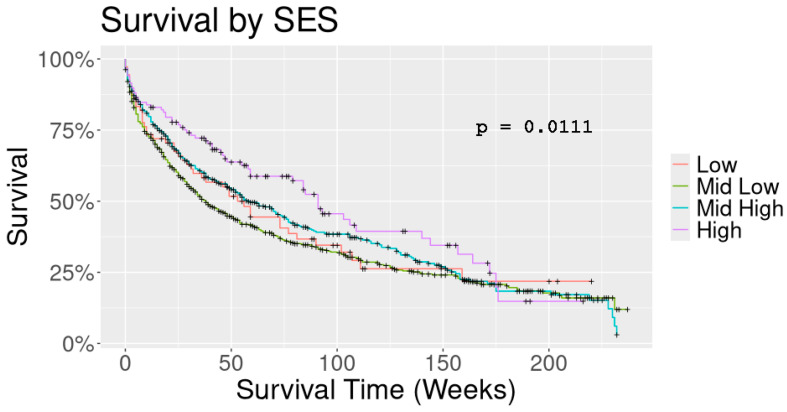
K-M and SES.

When evaluating the impact of SES on race, stage, age at diagnosis, and OS [Table 4], we noted marked differences between the races (as mentioned earlier, *p* < 0.0001); however, stage at diagnosis was unrelated to SES (*p* = 0.066). Age at diagnosis by itself was significantly different between the SES strata (*p* = 0.024). A Kaplan–Meier analysis showed that the OS was significantly better in the higher SES (*p* = 0.0111) group [Figure 1]. To dissect any confounding factors, we performed a multi-factorial Cox proportional hazard analysis to gauge the effect of the various factors on the overall risk of dying [Table 5]. The results indicated that, as predicted, stage at the time of diagnosis and SES contribute to the risk of dying, with individuals living in a high-SES zip code having a 45% reduced risk (HR 0.547) compared to people living in a low-SES zip code.

## 5. Discussion

Environmental and behavioral risk factors contribute to the development of cancer and serve as the foundation for preventive strategies. Despite considerable progress in medicine and surgery, the ultimate cure for cancer remains elusive. However, since cancer is a complex disease affected by modifiable risk factors, there is widespread agreement that preventative measures need to be implemented in cancer care. However, one of the challenges is to develop strategies that would influence the most vulnerable individuals. Therefore, the strategy needs to consist of (1) identifying vulnerable populations that might benefit the most from the implementation of preventative measures and (2) effectively utilizing the limited resources of regional medical centers to address these disparities. From a practical standpoint, based on the results of our study, we believe that, to address the inequities in CRC outcomes, the following should be further explored: 1. the use of community-embedded healthcare workers with a CRC-specific education program; and 2. the use of geocoding to target areas of low SES. In other words, we believe that appropriate resources should be made available from Federal and State Agencies to support the deployment of professionals like Nurse Navigators or Nurse Coordinators whose objective is to educate people residing in areas with a low SES on the importance of screening for CRC and the more favorable outcomes from the early diagnosis of CRC (i.e., more years of life). Mitigating previous negative experiences with healthcare delivery systems, addressing proper nutrition, abating language barriers, and providing financial support to offset the loss incurred in taking time off from work to seek medical care are a few other examples of how the optimization of resources could have a positive impact on the outcomes of CRC. These measures, in turn, have the potential to equalize the vulnerability of patient populations to CRC over time. In fact, embedding community-based healthcare workers to serve as patient navigators is seen as an effective and critical strategy [1]. In addition, the systematic screening of electronic medical records to identify potential subjects that would qualify for preventative service plays a role [15,16].

While the use of nationwide databases (SEER) serves an important role in unraveling the epidemiological traits of CRC, a more granular and focused approach that could be used by investigators [14,17,18,19] is to analyze the combination of SES with zip codes or other similar surrogates.

Our service area includes both urban and suburban populations; it should be noted that our sample is different from a nationwide sample (US) as it includes >35% non-whites, making our analysis more relevant to our geography and allowing us to target specific interventions. Moreover, despite the fact that there was a significant association between race and SES (Table 4), we could not demonstrate that race was a contributing factor in overall survival, perhaps indicating that race was a confounding variable in our dataset.

Many elegant analyses have shown how certain behaviors observed in people with a low SES can promote cancer. One such example is smoking and lung cancer: smoking, especially the use of mint-flavored tobacco, is more prevalent in AA and is a known risk factor for lung cancer [20]. Another example that links cancer to poverty is malnutrition, where obesity is a recognized risk factor for pancreatic cancer [21]. Of note, people with a low SES tend to have an unbalanced diet rich in saturated fat because of the low cost associated with such food. It is also known that certain races are more likely to live in areas of low SES, namely AA and Hispanics; more importantly, the observed and reported risk factors are preventable and modifiable with proper resources and education [21]. As an example, one might argue that, if we consider the case of pancreatic cancer mentioned above, the “obesity” risk factor could be controlled by providing adequate resources and education to poorer populations to promote a nutritious diet and proper exercise, or bariatric surgery when indicated. Notably, these examples suggest that the data supporting the association between low SES and race are strong, indicating a higher risk of developing cancer [22]. The results of our study support the notion that a low SES is associated with a higher risk of a poorer OS in CRC and a later diagnosis. In other words, racial background does not make an individual prone to developing colorectal cancer; however, a low SES is a risk factor for CRC. Poorer people are diagnosed later in life than the rest of the population and have worse outcomes when they are diagnosed with CRC. Patients in the low-SES group lack the basic means and education to achieve an early diagnosis and an optimal outcome, and uninsured patients do not have access to the safety net of hospitals or community cancer centers committed to the well-being of citizens. In this scenario, even a relatively well-educated individual might be denied access to care. A lack of transportation is a major impediment for poorer patients who are unable to be screened, diagnosed, or treated simply because they do not have “a ride” to reach the doctor’s office or the hospital. Individuals who lack documentation are reticent to see doctors for fear that volunteered information could lead to deportation. It is well established that screening for colorectal cancer saves life [23]. Education plays a major role in understanding the importance of colorectal cancer screening. Based on the above, we believe that our study has shown that poverty makes people more vulnerable to developing CRC and that this population has worse outcomes once diagnosed with the disease. We recognize that our results should be interpreted cautiously as patients’ deaths were likely from any cause, and people belonging to low-SES groups tend to have additional comorbidities. As such, the fact that they have lower survival could be, at least partially, due to other factors that are confounded in our study. However, given the aggressive nature of CRC, the trends observed in our data should be considered to be significant despite the influence of the confounding factors mentioned. Furthermore, we recognize that all registries and databases have inherent limitations, which, along with the retrospective nature of our analysis, represent a weakness of our study. However, such studies can still yield meaningful, albeit suggestive, trends despite potential issues with hidden variables [24]. We tried to ameliorate the effect of such factors by using advanced statistical techniques, such as covariate adjustment [25]. Despite the fact that our study may not meet the rigorous standards of clinical trials, it certainly raises important questions that warrant further investigation through carefully planned studies to unlock the relationship between CRC, SES, and geocoding.

## 6. Conclusions

In conclusion, our data suggest that the OS of patients with CRC is in part attributable to their zip code and its associated SES; race or ethnicity did not seem to be associated with overall survival, prompting the conclusion that geography (zip code) and its associated income level form major determinants of OS after being diagnosed with CRC. Others have also recognized the importance of the social determinants of health in CRC [12]. Unlike large academic medical centers (AMCs), which play a global role in the discovery of new science and the advancement of medicine and surgery, regional medical centers (RMCs) have a stake in the clinical well-being of their surrounding community and have important local responsibilities. In fact, many of the barriers listed in the discussion section of this paper are commonly overcome in community cancer centers. Therefore, more Federal and State resources should be programmatically directed to community cancer centers, and any other agencies that support research on and the screening and treatment of people of low SES diagnosed with CRC.

## Figures and Tables

**Table 1:** Demographics.

Variable	Value	Count (% of Total)
Race/Ethnicity	White	786 (61.9)
	African American	388 (30.6)
	Asian	32 (2.5)
	Hispanic	63 (5.0)
Sex	Male	610 (48.1)
	Female	659 (51.9)
Insurance	Private or Medicare	1115 (87.9)
	Medicaid	58 (4.6)
	Not insured	96 (7.6)
SES *	Low	73 (5.8)
	Mid-Low	621 (49.3)
	Mid-High	446 (35.4)
	High	119 (9.5)
Histopathology	Adenocarcinoma	1206 (95)
	Carcinoma	24 (1.9)
	Neuroendocrine carcinoma	38 (3)
Stage	Stage 0	17 (1.3)
	Stage 1	234 (18.4)
	Stage 2	318 (25.1)
	Stage 3	285 (22.5)
	Stage 4	258 (20.3)
Chemotherapy	Yes	535 (42.2)
	No	727 (57.3)
Surgery	Yes	1061 (83.6)
	No	207 (16.3)
Radiation therapy	Yes	189 (14.9)
	No	1078 (84.9)
Type Recurrence	Residual	302 (23.8)
	Local	29 (2.3)
	Metastatic disease	112 (8.8)
	None (disease free)	421 (33.2)
Status	Alive (based on latest visit to CHS)	447 (35.2)
	Dead	822 (64.8)
Average age at diagnosis	Mean ± st. dev.	68.7 ± 14.3
Overall Survival (months)	Median (IQR)	35.0 (11.86)

* Low = <60%; mid-low = between 60 and 100%; mid-high = between 100 and 140%; and high = >140% of median household income of entire NJ state.

**Table 2:** Zip code and SES.

Zip	No. Patients	Median Income (USD)	Median Cutoff SES
8618	244	44,657	Mid-Low
8638	179	54,044	Mid-Low
8648	136	85,380	Mid-High
8611	81	42,358	Mid-Low
8628	75	76,676	Mid-High
8610	73	62,112	Mid-Low
8619	61	74,215	Mid-High
8609	57	31,119	Low
8534	35	146,532	High
8690	33	91,870	Mid-High
8629	27	55,083	Mid-Low
8505	25	80,391	Mid-High
8620	24	92,261	Mid-High
8560	21	126,500	High
8022	16	78,139	Mid-High
8540	15	122,921	High
8530	12	82,331	Mid-High
8520	11	90,268	Mid-High
8608	10	13,150	Low

**Table 3:** Race and outcomes.

	Race				
Variable	Caucasian	Black	Asian	Hispanic	*p* Value
n	786	388	32	63	
Stage (%)					0.325
Stage 0	13 (1.9)	2 (0.6)	1 (3.3)	1 (1.8)	
Stage 1	150 (21.6)	64 (19.5)	9 (30.0)	11 (19.3)	
Stage 2	206 (29.6)	91 (27.7)	5 (16.7)	16 (28.1)	
Stage 3	171 (24.6)	84 (25.5)	11 (36.7)	19 (33.3)	
Stage 4	156 (22.4)	88 (26.7)	4 (13.3)	10 (17.5)	
SES (%)					<0.001
Low	20 (2.6)	39 (10.2)	3 (9.4)	11 (17.5)	
Mid-Low	273 (34.9)	300 (78.7)	4 (12.5)	44 (69.8)	
Mid-High	385 (49.2)	39 (10.2)	15 (46.9)	7 (11.1)	
High	105 (13.4)	3 (0.8)	10 (31.2)	1 (1.6)	
Insurance (%)					<0.001
Private or Medicare	734 (93.4)	311 (80.2)	28 (87.5)	42 (66.7)	
Medicaid	20 (2.5)	34 (8.8)	0 (0.0)	4 (6.3)	
Not insured	32 (4.1)	43 (11.1)	4 (12.5)	17 (27.0)	
Surgery (%)	125 (15.9)	73 (18.8)	2 (6.2)	7 (11.1)	0.140
Chemotherapy (%)	462 (59.2)	225 (58.1)	12 (37.5)	28 (45.2)	0.018
Age at diagnosis (years)	70.9 (14.1)	66.5 (13.7)	61.7 (10.9)	58.5 (14.9)	<0.001
Overall Survival (months)	33 (11.85)	35 (10.95)	29 (15.65)	46 (26.76)	0.567

**Table 4:** SES and outcomes.

	Socio-Economic Status (Median Cutoff)				
Variable	Low	Mid-Low	Mid-High	High	*p*-Value
n	73	621	446	119	
Race					<0.001
Caucasian	20 (27.4)	273 (44.0)	385 (86.3)	105 (88.2)	
Black	39 (53.4)	300 (48.3)	39 (8.7)	3 (2.5)	
Asian	3 (4.1)	4 (0.6)	15 (3.4)	10 (8.4)	
Hispanic	11 (15.1)	44 (7.1)	7 (1.6)	1 (0.8)	
Stage					0.066
Stage 0	0 (0.0)	6 (1.1)	5 (1.2)	6 (5.7)	
Stage 1	9 (16.4)	102 (19.1)	99 (24.1)	22 (21.0)	
Stage 2	17 (30.9)	155 (29.0)	115 (28.0)	28 (26.7)	
Stage 3	13 (23.6)	140 (26.2)	101 (24.6)	29 (27.6)	
Stage 4	16 (29.1)	131 (24.5)	90 (22.0)	20 (19.0)	
Insurance (%)					<0.001
Private or Medicare	60 (82.2)	514 (82.8)	418 (93.7)	116 (97.5)	
Medicaid	6 (8.2)	37 (6.0)	12 (2.7)	1 (0.8)	
Not insured	7 (9.6)	70 (11.3)	16 (3.6)	2 (1.7)	
Surgery (%)	11 (15.1)	118 (19.0)	61 (13.7)	15 (12.6)	0.075
Chemotherapy (%)	42 (58.3)	354 (57.3)	251 (56.7)	73 (61.3)	0.830
Age at Diagnosis (years)	65.1 (12.9)	68.3 (14.2)	70.1 (14.2)	68.9 (15.9)	0.024
Overall Survival (months)	40.5 (10.88)	30.0 (8.79)	38 (13.87)	47 (22.91)	0.020

**Table 5:** Risk of dying: multifactorial regression analysis.

Parameter		Chi-Square	*p* Value	Hazard Ratio	95% Hazard Ratio Confidence Limits	
Stage *	Stage 1	1.98	0.1599	1.914	0.774	4.734
	Stage 2	7.69	0.0056	3.560	1.451	8.733
	Stage 3	13.35	0.0003	5.418	2.188	13.413
	Stage 4	44.28	<0.0001	22.127	8.888	55.084
Race/Ethnicity *	Asian	6.28	0.0122	2.016	1.165	3.489
	Black	0.12	0.7323	0.968	0.802	1.168
	Hispanic	0.47	0.4931	0.861	0.561	1.321
SES *	High	6.73	0.0095	0.547	0.347	0.863
	Mid-High	1.16	0.2806	0.812	0.556	1.185
	Mid-Low	0.11	0.7425	1.062	0.743	1.516
Insurance *	Medicaid	9.46	0.0021	1.742	1.223	2.481
	Not Insured	0.14	0.7089	1.063	0.772	1.462
Surgery		50.07	<0.0001	0.420	0.330	0.534
Chemotherapy		43.68	<0.0001	0.520	0.428	0.631
Age at Diagnosis		100.23	<0.0001	1.038	1.030	1.045

* Reference groups by variable: stage = 0; race or ethnicity = Caucasian; SES = low; insurance = private/Medicare.

## Data Availability

All relevant data are within the paper.

## Abbreviations

AA	African Americans
CRC	colorectal cancer
PAOI	physical area of intervention
OS	overall survival
SES	socio-economic status
SEER	surveillance, epidemiology, and end results program
